# Gut microbiota variations in patients diagnosed with major depressive disorder—A systematic review

**DOI:** 10.1002/brb3.2177

**Published:** 2021-05-28

**Authors:** Julie Kristine Knudsen, Caspar Bundgaard‐Nielsen, Simon Hjerrild, René Ernst Nielsen, Peter Leutscher, Suzette Sørensen

**Affiliations:** ^1^ Centre for Clinical Research North Denmark Regional Hospital Hjoerring Denmark; ^2^ Department of Clinical Medicine Aalborg University Aalborg Denmark; ^3^ Psychosis Research Unit Aarhus University Hospital Aarhus Denmark; ^4^ Department of Clinical Medicine Aarhus University Aalborg Denmark; ^5^ Department of Psychiatry Aalborg University Hospital Aalborg Denmark

**Keywords:** depression, gut‐brain axis, major depressive disorder, microbiome, microbiota, unipolar depression

## Abstract

**Objective:**

The etiology of major depressive disorder (MDD) is multi‐factorial and has been associated with a perturbed gut microbiota. Thus, it is therefore of great importance to determine any variations in gut microbiota in patients with MDD.

**Methods:**

A systematic literature search was conducted including original research articles based on gut microbiota studies performed in patients with MDD. Demographic and clinical characteristics, applied methodology and observed gut microbiota composition were compared between included studies.

**Results:**

Seventeen studies were included with a total of 738 patients with MDD and 782 healthy controls using different DNA purification methods, sequencing platforms and data analysis models. Four studies found a reduced α‐diversity in patients with MDD, while gut microbiota compositions clustered separately according to β‐diversity between patients and controls in twelve studies. Additionally, there was an increase in relative abundance of *Eggerthella, Atopobium,* and *Bifidobacterium* and a decreased relative abundance of *Faecalibacterium* in patients with MDD compared with healthy controls.

**Conclusion:**

Gut microbiota differs significantly when comparing patients with MDD and healthy controls, though inconsistently across studies. The heterogeneity in gut microbiota compositions between the studies may be explained by variations in study design.

## Graphical abstract

The gut microbiota is significantly different in patients diagnosed with depression compared to healthy controls.

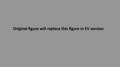

## 1 INTRODUCTION

The etiology of major depressive disorder (MDD) is characterized by complex interactions between genetic, biological, and environmental factors (Saveanu & Nemeroff, 2012). Patients with MDD often have gastrointestinal disturbances, such as abdominal pain, cramping, bloating, diarrhea, and/or constipation (Walker et al., 1992). A direct causative link between the gastrointestinal disturbances and MDD has not yet been established, but the gut microbiota has been suggested to be involved, though its role not yet fully elucidated (Bastiaanssen et al., 2020; Capuco et al., 2020; Carlessi et al., 2019; Caspani et al., 2019; Clarke, 2020; Cruz‐Pereira et al., 2020; Dinan & Cryan, 2019; Du et al., 2020; Kelly et al., 2019; Simpson et al., 2020). Gut microbiota has suggested to play a role in the bidirectional communication between the gastrointestinal system and the brain, also known as the gut‐brain axis (Capuco et al., 2020; Cryan & Dinan, 2012). The study by Sudo et al. indicated an association between gastrointestinal bacteria and altered behavior (Sudo et al., 2004). In this study, colonization of germ‐free mice with *Escherichia coli* resulted in a significantly altered behavior when exposed to stress compared to specific pathogen‐free mice. The altered behavior in the mice was associated with a pro‐inflammatory profile and inoculation with a strain of *Bifidobacterium* attenuated both behavioral and immunological changes. This experiment displayed how a negative behavioral effect can be produced by a specific bacterial pathogen, but also rescued by introduction of a beneficial species.

Other studies have shown that gut microbes interact with the central nervous system by signaling through bacterial components recognized by the immune system (Schroeder & Backhed, 2016), or by various derived metabolites (Fischbach & Segre, 2016). A study in rats showed that intravenous administration of lipopolysaccharide (LPS), a cell wall component of gram‐negative bacteria, induced a depression‐like phenotype as well as increased levels of white blood cells and pro‐inflammatory cytokines (Wrotek et al., 2016). Furthermore, increased plasma concentrations of IgM and IgA antibodies against Gram‐negative enterobacteria were observed in patients with MDD (Maes et al., 2012). Meta‐analyses have reported low‐grade systemic inflammation in patients with MDD, indicated by higher levels of C‐reactive protein (Osimo et al., 2019) and pro‐inflammatory cytokines (Osimo et al., 2020). Importantly, patients diagnosed with MDD have a dysfunctional microbiota‐host signaling and interactions (Cruz‐Pereira et al., 2020) characterized by increased plasma cortisol levels (Furtado & Katzman, 2015; Otte et al., 2016) and T‐cell dysregulation (Beurel & Lowell, 2017; Cruz‐Pereira et al., 2020; Furtado & Katzman, 2015; Otte et al., 2016).

Not all metabolites produced by the gut microbiota have detrimental effects on emotional and cognitive functions. Short‐chain fatty acids (SCFAs), produced by beneficial species such as *Faecalibacterium* from indigestible fibers, can induce vagus nerve stimulation in the colon (Cawthon & Serre, 2018; Chun et al., 2016; Schroeder & Backhed, 2016), microglia maturation and activation (Sharon et al., 2016; Yang & Chiu, 2017), as well as produce brain‐derived neurotrophic factor (BDNF) (Sandberg et al., 2018), a neural growth hormone. In clinical trials, healthy volunteers consuming probiotic supplements, of which several species produce SCFAs, experienced improved cognition (Marotta et al., 2019; Tillisch et al., 2013) or mood (Benton et al., 2007; Marotta et al., 2019). Combined, these experiments suggest an association between gastrointestinal bacterial species and behavioral alterations, which may become potential therapeutic targets for MDD treatment.

Recently, several clinical studies have explored the association between a specific composition of gut microbiota and depressive features (Barandouzi et al., 2020; Cheung et al., 2019; Sanada et al., 2020). In the assessment of gut microbiota, however, the study design itself may affect the overall gut microbiota composition. Diet has been found to have a major impact on bacterial species in the gut microbiota (Conlon & Bird, 2014). In the experimental processing, choice of DNA purification methods (Costea et al., 2017) and primers targeting the 16S rRNA gene for sequencing (Albertsen et al., 2015; Hamady & Knight, 2009; Lozupone et al., 2013) can considerably affect the observed composition of the gut microbiota. In determining if the gut microbiota is altered in patients with MDD, it is imperative to assess the methods used to characterize the gut microbiota.

The aim of this review was to evaluate and compare studies of gut microbiota composition in patients with MDD compared with healthy controls.

## 2 METHODS

A protocol for this systematic review was uploaded and accepted into the (PROSPERO) server under the ID number CRD42018104925.

### 2.1 Information sources

The databases PubMed, Embase (Ovid), and PsycINFO (Ovid) were searched for articles published up until November 13th, 2020. The literature search was conducted according to the Preferred Reporting Items for Systematic Reviews and Meta‐analyses (PRISMA) guidelines (Moher et al., 2009). The search strategies are described Supporting information 1. All fields (including title and abstract) were explored to ensure completeness of the literature search.

### 2.2 Inclusion and exclusion criteria

Articles were included if they met the following inclusion criteria:

- Clinical studies performed on patients diagnosed with MDD according to criteria in the Diagnostic and Statistical Manual of Mental Disorders (DSM) or the International Classification of Diseases (ICD).
- Assessment of the gut microbiota composition through genomic analysis, including both targeted and nontargeted approaches.
- Inclusion of a control group of nondepressed individuals.

Studies were excluded if they met the following exclusion criteria:

- The focus of the study was inclusion of patients with known comorbidities, such as assessing the gut microbiota in patients with both MDD and inflammatory bowel disorders.
- Assessment of the effect of pro‐, pre‐, syn‐ or antibiotics on a group of patients with MDD with no baseline measurement of the original gut microbiota.

### 2.3 Study selection

Studies identified in the systematic literature search were imported into the EndNote software (Clarivate Analytics) for removal of duplicates. Files generated from these databases were imported into the Systematic Review Facility (SyRF) app (http://app.syrf.org.uk). SyRF was then used for screening of papers and data were extracted manually. Authors JKK and CBN independently reviewed and selected studies based on title and abstract presented by the SyRF app, and later manually reviewing the full‐text. Both reviewers consistently agreed upon which studies to include and there was thus no need to include a third reviewer. JKK extracted outcome measures as described below.

### 2.4 Outcome measures

Demographic and clinical data were extracted from the patient and control groups. Sample processing and analyses were focused on the methods applied to analyze the bacterial community: fecal storage conditions, DNA extraction process, choice of primers, and platforms for DNA sequencing, bioinformatics analysis programs, and databases used for taxonomic classification. The gut microbiota composition results of each study were extracted and focused on α‐ and β‐diversity measures and overall significant differences in composition between the two groups. Significantly altered bacteria was included for taxa phylum, family and genus level, only.

## 3 RESULTS

**FIGURE 1:**
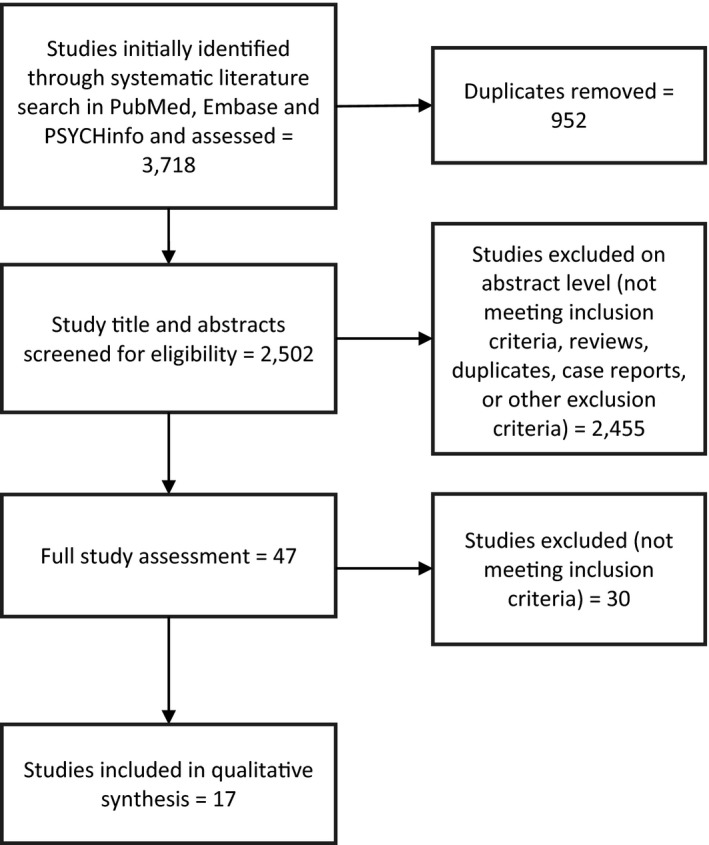
PRISMA flow diagram for article selection. Three databases were used to identify relevant papers, and SyRF was used to screen abstracts

Screening of articles was performed according to PRISMA guidelines (Figure 1). The literature search identified 3,718 articles, of which 3,701 were subsequently excluded. Thus, this left seventeen articles for further analysis (Aizawa et al., 2016; Chen et al., 2018, 2020; Chung et al., 2019; Huang et al., 2018; Jiang et al., 2015; Kelly et al., 2016; Lai et al., 2019; Lin et al., 2017; Liu et al., 2020; Mason et al., 2020; Naseribafrouei et al., 2014; Rong et al., 2019; Stevens et al., 2020; Vinberg et al., 2019; Zheng et al., 2016, 2020).

### 3.1 Clinical information reveals highly heterogeneous study populations

Sixteen of the included studies were designed as cross‐sectional, case‐control studies comparing the gut microbiota of patients with MDD and healthy controls. One study was a longitudinal study, where each participant provided fecal samples at three different time points (Lin et al., 2017). The seventeen studies included a total of 738 patients diagnosed with MDD and 782 healthy controls (Table 1).

**TABLE 1:** Demographic characteristics

Demographic data of patients with MDD and healthy controls	Participants		Age mean (*SD*)	Male (%)	Education (%)	Employed (%)
Naseribafrouei et al. (Naseribafrouei et al., 2014)					Years of education	
	Controls	*n* = 18	46.1 (13.9)	39	13.5	
	Patients	*n* = 37	49.2 (13.9)	46	12.7	
Jiang et al. (Jiang et al., 2015)					High school level (%)	
	Controls	*n* = 30	26.8 (5.4)	50	43	13
	A‐MDD	*n* = 29	25.3 (5.4)	62	31	28
	R‐MDD	*n* = 17	27.1 (5.4)	53	24	18
Zheng et al. (2016) (Zheng et al., 2016)					Years of education	
	Controls	*n* = 63	41.8 (12.3)	37		
	Patients	*n* = 58	40.5 (11.7)	38		
Aizawa et al. (Aizawa et al., 2016)					Degree level (%)	
	Controls	*n* = 43	42.8 (12.7)	58	15.3	
	Patients	*n* = 57	39.4 (10.0)	39	15.2	
Kelly et al. (Kelly et al., 2016)					Years of education	
	Controls	*n* = 33	45.8 (11.9)	58	79	94
	Patients	*n* = 34	45.8 (11.5)	62	24	47
Lin et al. (Lin et al., 2017)						
	Controls	*n* = 10	38.1 (2.9)	60	13.8	
Patients	*n* = 10	36.2 (10.1)	60	15.3	
Chen et al. (2018) (Chen et al., 2018)						
	Controls	*n* = 44	42.8 (15.1) (M) 43.9 (12.1) (F)	45		
Patients	*n* = 44	40.1 (11.1) (M) 41.5 (11.5) (F)	45		
Rong et al. (Rong et al., 2019)						
	Controls	*n* = 30	38.5 (10.2)	47		
Patients	*n* = 30	41.6 (10.4)	71		
Chung et al. (Chung et al., 2019)					Years of education	
	Controls	*n* = 36	41.2 (12.7)	38	15.8 (2.2)	100
	Patients	*n* = 37	45.8 (14.1)	18	13.8 (3.2)	67
Huang et al. (Huang et al., 2018)						
	Controls	*n* = 27	42.3 (14.1)	26		
Patients	*n* = 27	48.7 (12.8)	26		
Vinberg et al. (Vinberg et al., 2019)						
	Low‐risk	*n* = 22	37.2 (7.7)	28		
	High‐risk	*n* = 32	38.2 (9.4)	28		
Affected	*n* = 45	37.7 (8.9)	25		
Chen et al. (2020) (Chen et al., 2020)						
	Controls	*n* = 27 (Y) *n* = 44 (M)	25.0 (2.3) (Y) 47.2 (8.1) (M)	30 (Y) 23 (M)		
Patients	*n* = 25 (Y) *n* = 45 (M)	24.0 (3.7) (Y) 45.0 (7.8) (M)	28 (Y) 31 (M)		
Lai et al. (Lai et al., 2019)						
	Controls	*n* = 29	39.4 (11.0)	45		
Patients	*n* = 26	43.7 (11.5)	31		
Lai et al. (Lai et al., 2019)						
	Controls	*n* = 47	21.7 (2.1)	27		
Patients	*n* = 43	22.7 (1.8)	15		
Mason et al. (Mason et al., 2020)						
	Controls	*n* = 10	33.0 (8.4)	40		
Patients	*n* = 14	41.9 (12.0)	21		
Stevens et al. (Stevens et al., 2020)						
	Controls	*n* = 20		50		
Patients	*n* = 20		30		
Zheng et al. (2020) (Zheng et al., 2020)						
	Controls	*n* = 171 (D) *n* = 46 (V)	27.9 (5.5) (D) 45.5 (7.1) (V)	42 (D) 52 (V)		
Patients	*n* = 122 (D) *n* = 43 (V)	26.5 (4.1) (D) 37.1 (9.2) (V)	37 (D) 33 (V)		

Note

Data are presented as mean (Standard deviation, *SD*) or percentage. Several studies assessed subgroups, which are designated in the table; A‐MDD, treatment‐resistant patients with MDD; R‐MDD, Patients with MDD responding to antidepressant medical treatment; M, Male; F, Female; Low‐ and High‐risk, Healthy twin siblings with assessed genetic risk of MDD; Y, Young; MA, Middle‐aged; D, Discovery population; V, Validation population. BMI: Body Mass Index.

In the majority of the studies, patients were assessed according to the DSM criteria (Aizawa et al., 2016; American Psychiatric Association, 1994, 2000; Chen et al., 2018, 2020; Chung et al., 2019; Jiang et al., 2015; Kelly et al., 2016; Lai et al., 2019; Lin et al., 2017; Liu et al., 2020; Mason et al., 2020; Rong et al., 2019; Stevens et al., 2020; Zheng et al., 2016); the remaining studies used the ICD criteria (Hiller et al., 1994; Huang et al., 2018; Naseribafrouei et al., 2014; Vinberg et al., 2019) (Table 2). Eight of the studies excluded patients with comorbid psychiatric disorders (Chen et al., 2018, 2020; Chung et al., 2019; Jiang et al., 2015; Lai et al., 2019; Mason et al., 2020; Rong et al., 2019; Zheng et al., 2020), while six of the studies excluded patients with inflammatory bowel disorders (Chen et al., 2018; Huang et al., 2018; Jiang et al., 2015; Kelly et al., 2016; Vinberg et al., 2019; Zheng et al., 2020). Otherwise, there was no consensus between studies on exclusion criteria for specific psychiatric or somatic disorders. Four studies also examined a subset of patients with either bipolar disorder (Rong et al., 2019; Vinberg et al., 2019; Zheng et al., 2020) or comorbid anxiety (Mason et al., 2020). Data from these subgroups are not included in this review. Some studies included antidepressant treatment‐naïve patients exclusively (Chen et al., 2018; Huang et al., 2018), or a subset of patients not previously prescribed antidepressants (Naseribafrouei et al., 2014; Zheng et al., 2016, 2020). In the remaining studies, the use and description of pharmacological treatment varied, with only a few studies limiting their patients to specific pharmacological treatment (Chung et al., 2019; Lin et al., 2017). In conclusion, participant demographics and clinical characteristics varied between the studies, thus limiting comparability and generalizability.

**TABLE 2:** Clinical information

Clinical information about patients with MDD and healthy controls	Participants		HDRS	MADRS mean (*SD*)	BDI mean (*SD*)	BMI mean (*SD*)	Antidepressant treatment
Naseribafrouei et al. (Naseribafrouei et al., 2014)							Antidepressant treatment, mean (*SD*)
	Controls	*n* = 18		7.2 (4.8)		24.7 (3.3)	0.1 (0.2)
	Patients	*n* = 37		26.3 (7.6)		25.9 (4.2)	0.7 (0.5)
			HAMD−24 mean (*SD*)				SSRIs or SNRIs treatment, No. (%)
Jiang et al. (Jiang et al., 2015)	Controls	*n* = 30	NA	NA		19.6 (3.4)	0
	A‐MDD	*n* = 29	29.8 (7.6)	27.4 (8.5)		20.3 (3.4)	21 (72)
	R‐MDD	*n* = 17	8.3 (4.6)	6.9 (4.3)		21.8 (3.4)	17 (100)
Zheng et al. (2016) (Zheng et al., 2016)			HAMD−21 mean (*SD*)				Antidepressant treatment, No. (%)
	Controls	*n* = 63	0.3 (0.7)			22.6 (2.5)	0
	Patients	*n* = 58	22.8 (4.4)			22.0 (2.4)	19 (33)
Aizawa et al. (Aizawa et al., 2016)			HAMD−21 mean (*SD*)				Imipramine equivalent, dose conversion (*SD*)
	Controls	*n* = 43	NA			22.3 (3.7)	NA
	Patients	*n* = 57	16.9 (6.8)			23.2 (3.6)	187.7 (152.7)
Kelly et al. (Kelly et al., 2016)			HAMD−17 median (range)				SSRIs treatment, No. (%)
	Controls	*n* = 33	NA		NA	24.6 (2.7)	0
	Patients	*n* = 34	19.5 (14)		32.4 (9.9)	26.2 (4.5)	34 (100)
Lin et al. (Lin et al., 2017)			HAMD−17				Escitalopram daily, No. (%)
	Controls	*n* = 10	NA			24.2 (2.0)	0
	Patients	*n* = 10	>23			23.8 (1.9)	37 (100)
Chen et al. (2018) (Chen et al., 2018)			HAMD−17 median (range)				
	Controls	*n* = 44	NA			22.5 (2.3) (M) 22.6 (2.4) (F)	NA
Patients	*n* = 44	23.9 (3.7)			22.2 (2.2) (M) 22.0 (2.2) (F)	0
Rong et al. (Rong et al., 2019)			HAMD−17 mean (*SD*)				Antidepressant treatment, No. (%)
	Controls	*n* = 30	NA			22.0 (3.2)	NA
	Patients	*n* = 30	20.4 (3.4)			21.5 (2.1)	23 (74.2)
Chung et al. (Chung et al., 2019)							Escitalopram 5−20mg daily
	Controls	*n* = 36			4.5 (4.9)	24.0 (3.9)	0
	Patients	*n* = 37			19.2 (12.5)	22.8 (4.2)	31 (86.1)
Huang et al. (Huang et al., 2018)							Antidepressant treatment
	Controls	*n* = 27	NA			23.4 (2.9)	NA
	Patients	*n* = 27				23.8 (2.8)	0
Vinberg et al. (Vinberg et al., 2019)			HAMD−17 mean (*SD*)				Antidepressant treatment, No. (%)
	Low‐risk	*n* = 22	2.4 (2.4)			24.5 (3.1)	NA
	High‐risk	*n* = 32	2.7 (2.5)			23.9 (3.1)	NA
	Affected	*n* = 71	4.9 (3.9)			26.5 (7.0)	49 (69)
Chen et al. (2020) (Chen et al., 2020)			HDRS mean (*SD*)				
	Controls	*n* = 27 (Y) *n* = 44 (M)	0.3 (0.6) (Y) 0.3 (0.7) (M)			21.5 (2.4) (Y) 23.2 (2.3) (M)	
	Patients	*n* = 25 (Y) *n* = 45 (M)	22.6 (3.2) (Y) 23 (4.6) (M)			22.1 (2.2) (Y) 22.6 (2.6) (M)	
Lai et al. (Lai et al., 2019)			HAMD−17 mean (*SD*)				Antidepressant treatment, %
	Controls	*n* = 29	NA			21.1 (2.2)	NA
	Patients	*n* = 26	19.8 (3.0)			21.2 (2.2)	81
Liu et al. (Liu et al., 2020)							Antidepressant treatment, %
	Controls	*n* = 47					2
	Patients	*n* = 43					65
Mason et al. (Mason et al., 2020)							Antidepressant treatment, %
	Controls	*n* = 10				25.6 (3.5)	0
	Patients	*n* = 14				31.0 (5.8)	64
Stevens et al. (Stevens et al., 2020)							Antidepressant treatment, %
	Controls	*n* = 20					NA
	Patients	*n* = 20					75
Zheng et al. (2020) (Zheng et al., 2020)			HDRS mean (*SD*)				Antidepressant treatment, %
	Controls	*n* = 171 (D) *n* = 46 (V)	NA			22.1 (3.4) (D) 22.1 (2.5) (V)	NA
	Patients	*n* = 122 (D) *n* = 43 (V)	22.7 (5.5) (D) 23.5 (4.6) (V)			22.4 (3.3) (D) 22.1(3.1) (V)	42 (D)

Note

Data are presented as mean (standard deviation, *SD*), median (range) or number of participants. Severity of MDD was measured by validated scales; Hamilton Depression Rating Scale (HDRS), Beck Depression Inventory (BDI) and Montgomery–Åsberg Depression Rating Scale (MADRS). HDRS was often applied as either the 17‐item questionnaire (HAMD‐17), the 21‐item questionnaire (HAMD‐21) or the 24‐item questionnaire (HAMD‐24). Several studies assessed subgroups; A‐MDD, treatment‐resistant patients with MDD; R‐MDD, Patients with MDD responding to antidepressant medical treatment; M, Male; F, Female; Low‐ and High‐risk, Healthy twin siblings with assessed genetic risk of MDD; Y, Young. MA: Middle‐aged; D, Discovery population; V, Validation population.

### 3.2 Methodology and bioinformatics analyses of gut microbiota composition varied considerably between studies

In all studies, fecal samples were collected to determine the gut microbiota composition of both patient and control groups (Table 3). The majority of the studies used DNA sequencing to assess the gut microbiota: thirteen performed 16S ribosomal ribonucleic acid (rRNA) gene sequencing (Janda & Abbott, 2007; Jovel et al., 2016) and two used shotgun sequencing (Jovel et al., 2016). The remaining two studies used targeted reverse transcriptase‐quantitative polymerase chain reaction (qRT‐PCR) with species‐specific primers (Aizawa et al., 2016) or a combination of 16S rRNA gene sequencing and qRT‐PCR (Mason et al., 2020).

**TABLE 3:** Sample preparation and processing in the seventeen studies assessed

Fecal sample preparation and processing, and bioinformatics analysis methods	Storage method	DNA/RNA extraction kit or method	Sequencing system	Target hypervariable region of the 16S rRNA gene (primer pair specification)	Bioinformatic analysis program	Database used for taxonomic classification
Naseribafrouei et al. (Naseribafrouei et al., 2014)	−70˚C.	Mag mini kit (LGC) + bead beating	MiSeq (Illumina)		k‐mer frequency based approach combined with QIIME	RDP
Jiang et al. (Jiang et al., 2015)	−80˚C	QIAamp DNA Stool Mini Kit (QIAGEN) + bead beating	GS FLX Titanium system (454 Life Science, Roche)	V1‐V3 (none)	Mothur v1.25.0	RDP
Zheng et al. (2016) (Zheng et al., 2016)	−80˚C	PowerSoil (MoBio)	V3‐V5: GS FLX Titanium system (454 Life Sciences, Roche)	V3‐V5 (none)	V3‐V5: Mothur v1.31.2	RDP
			V4‐V5: MiSeq (Illumina)	V4‐V5 (none)	V4‐V5: QIIME	
Aizawa et al. (Aizawa et al., 2016)	RNAlater at room temperature	Intestinal Flora‐SCAN (Yakult)		Primers targeting specific bacteria, see table legend for specification		Primers tested in RDP
Kelly et al. (Kelly et al., 2016)	21 samples: −80˚C	QIAamp DNA Stool Mini Kit (QIAGEN)	MiSeq (Illumina)		QIIME	SILVA v111
	43 samples: directly procesed					
Lin et al. (Lin et al., 2017)	−70˚C	Tiagen DNA Stool Mini Kit (Tiagen Biotech)	MiSeq (Illumina)	V3‐V4 (none)	Mothur v1.30	SILVA v119
Chen et al. (2018) (Chen et al., 2018)	−80˚C	PowerSoil (MoBio)	GS FLX Titanium system (454 Life Science, Roche)	V3‐V5 (none)	Mothur v1.31.2	RDP
Rong et al. (Rong et al., 2019)	−80˚C	StoolGen DNA kit (CWBiotech)	HiSeq (Illumina)	Shotgun sequencing	Rstudio	
Chung et al. (Chung et al., 2019)	Transported at 4˚C, stored at −80˚C	QIAamp DNA Stool Mini Kit (QIAGEN) OR phenol‐chloroform +bead beating	53 samples: MiSeq (Illumina)	53 samples: V3‐V4 (515F and 805R primers)	QIIME	Greengenes
			20 samples: MiniSeq (Illumina)	20 samples: V4 (515F and 806R primers)		
Huang et al. (Huang et al., 2018)	−80˚C	PowerSoil (MoBio)	HiSeq2500 (Illumina)	V3‐V4 (314F and 805R primers)	Demographic data: QIIME. Bioinformatics: Rstudio	Greengenes
Vinberg et al. (Vinberg et al., 2019)	Room temperature for 24−72h, then −80˚C	Nucleospin 96 Soil kit (Macherey‐Nagel) + bead beating	MiSeq (Illumina)	V3‐V4 (S‐D‐Bact−0,341‐b‐S−17 and S‐D‐Bact−0,785‐a‐A−21)	Mothur v1.38.1	RDP
Chen et al. (2020) (Chen et al., 2020)	Frozen	PowerSoil (MoBio)	454 Sequencing (454 Life Science, Roche)	V3‐V5	Mothur v1.31.2	RDP
Lai et al. (Lai et al., 2019)	−80˚C	StoolGen DNA kit (CWBiotech)	HiSeq2500 (Illumina)	Shotgun sequencing		MEGAN5
Liu et al. (Liu et al., 2020)	−80˚C	ZymoBIOMICS 96 DNA kit (Zymo Research)	MiSeq (Illumina)	V4 (515F and 806R primers)	QIIME2	SILVA v132
Mason et al. (Mason et al., 2020)	−80˚C	Phenol/cholorform/isoamyl alcohol extraction +bead beating	454 Titanium System (454 Life Science, Roche)	V4 (563F and 926BSR primers)	QIIME	SILVA
Stevens et al. (Stevens et al., 2020)	−80˚C	EZNA DNA Extraction kit (Omega Biotek)	MiSeq (Illumina)	V3‐V4 (314F and 805R primers)	Rstudio	SILVA v132
Zheng et al. (2020) (Zheng et al., 2020)	Frozen	OMEGA‐soil DNA kit (Omega Biotek)	MiSeq (Illumina)	V3‐V4 (338F and 806R primers)	Rstudio	RDP

Note

Sixteen of the studies used next generation gene sequencing, with either 16S rRNA gene sequencing or shotgun metagenomics sequencing for bacterial characterization. Two studies performed targeted qPCR; Aizawa et al. and Mason et al. Distinct species targeted in the study by Aizawa et al. by qRT‐qPCR were *Enterococcus spp., Staphylococcus spp. Enterobacteriaceae spp., Prevotella spp., Lactobacillus spp., Pseudomonas spp., Bifidobacterium spp., and Lactobacillus spp*. The authors additionally included six subspecies, *Clostridium spp*. and an additional subspecies, *Bacteroidetes fragilis* and the *Atopobium* cluster. Mason et al. performed qPCR with primers designed against *Eubacteria*, *Enterobacteriaceae*, *Eubacterium* *rectale/Clostridium cluster XIVa*, *Lactobacillus/Enterococcus group*, *Bacteroides*, and the *Clostridium leptum group*. QIIME, Quantitative Insights Into Microbial Ecology; RDP, Ribosomal Database Project.

The studies performing 16S rRNA gene analysis or metagenomic sequencing primarily applied one of three distinct sequencing platform (Illumina MiSeq, Illumina HiSeq, or 454 sequencing platforms), bioinformatics analysis pipelines (Mothur, QIIME, or RStudio) or taxonomic classification databases (Ribosomal Database Project, GreenGenes, or SILVA). However, there were substantial differences in methodology. Choice of nucleic acid purification kits varied extensively and the primers targeting the hypervariable regions of the 16S rRNA gene were different. In studies exploring the same region of interest, the primer constructs were not identical, except in two studies targeting the V4 region using the 515F/806R primer pair (Chung et al., 2019; Liu et al., 2020), or in two studies targeting the V3‐V4 region using the 314F/805R primer pair (Huang et al., 2018; Stevens et al., 2020). Aizawa et al. did not extract bacterial DNA, but rather RNA with the Intestinal Flora‐SCAN (Yakult). The Intestinal Flora‐SCAN targets the 16S or 23S rRNA sequences in a subset of bacteria using primer pairs blasted against the Ribosomal Database Project (Aizawa et al., 2016). Mason et al. likewise used targeted qPCR, but purified bacterial DNA and designed primers for distinct bacterial species, as well as used nontargeted 16S rRNA gene sequencing (Mason et al., 2020).

Overall, the studies were highly heterogeneous regarding choice of nucleic acid extraction method, 16S rRNA gene target region and bioinformatics analysis program, as well as database for taxonomic classification.

### 3.3 Both diversity and specific taxa were reported significantly different across studies

Despite methodological heterogeneity, the studies observed several variations in gut microbiota composition between patients and controls. Observations of differences in α‐ or β‐diversity indices between patients and controls are presented in Table 4. Generally, results on bacterial diversity differed extensively between studies. The majority of the studies did not find significant differences in α‐diversity, using several diversity indices (Chen et al., 2018, 2020; Chung et al., 2019; Jiang et al., 2015; Kelly et al., 2016; Lai et al., 2019; Liu et al., 2020; Mason et al., 2020; Naseribafrouei et al., 2014; Rong et al., 2019; Vinberg et al., 2019; Zheng et al., 2016, 2020). Some studies, however, observed a reduction in bacterial α‐diversity in patients with MDD compared with controls (Huang et al., 2018; Kelly et al., 2016; Rong et al., 2019; Vinberg et al., 2019), except in the study by Jiang et al., where an increased Shannon index was observed in patients defined as antidepressant treatment resistant (Jiang et al., 2015). Similarly, contradicting results were also found in the analyses of β‐diversity. Distinguishing between patients and healthy controls was possible in twelve of the seventeen studies using a variety of different analytical methods (Chen et al., 2018, 2020; Chung et al., 2019; Huang et al., 2018; Lai et al., 2019; Lin et al., 2017; Liu et al., 2020; Naseribafrouei et al., 2014; Stevens et al., 2020; Zheng et al., 2016, 2020). Overall, sixteen of the seventeen analyses found compositional differences using either α‐ or β‐diversity indices in the gut microbiota between patients and healthy controls despite methodological heterogeneity.

**TABLE 4:**
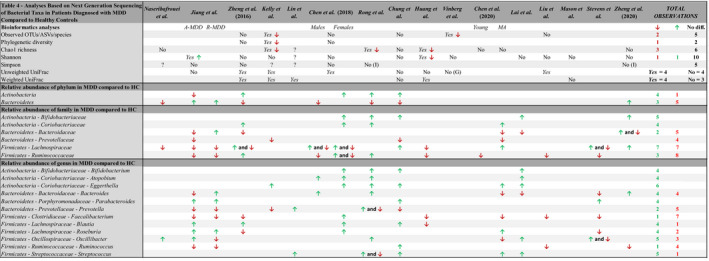
Bacterial taxa obtained from next generation sequencing of the bacterial DNA comparing patients with MDD and healthy controls

Note

Statistically significant differences observed between the patient and control group in the bioinformatical analyses are designated as either “*Yes*” or “No”. Arrows specify the direction of change. Fields with a question mark correspond to studies, where the bioinformatics analysis was conducted according to the methods section, but no conclusions were reported in the results section. Bacterial taxa are listed as *phylum ‐ family ‐ genus*. Arrows here specify the increase (green) or decrease (red) in relative abundance of bacterial taxa in patients compared with controls, if this was observed in four or more study populations. If a study conducted analyses on several populations, such as dividing participants into sexes, each observation made was counted as an independent observation. "Total observations" is the cumulative number of times a specific direction of change was observed across the studies. Taxa significantly different between patients and controls were presented, when four or more studies agreed upon a specific direction of change. A‐MDD: treatment‐resistant patients with MDD. R‐MDD: Patients with MDD responding to antidepressant medical treatment. MA: Middle‐aged. OTUs: Operational taxonomic Units. ASVs: Amplicon Sequence Variants. (I): Inverse Simpson. (G): Generalized UniFrac, a combination of weighted and unweighted UniFrac.

Although not all studies agreed upon a different composition of the gut microbiota between patients and controls, sixteen out of seventeen studies showed variations in relative abundance of individual taxa between cases and controls. A list of all bacterial taxa significantly different in relative abundance in patients compared with controls is presented in Supporting information 2. A selected fraction of these is also presented in Table 4. In total, 5 phyla, 36 families, and 78 genera of bacteria were found to be significantly altered in relative abundance between patients and healthy controls. At phylum level, Actinobacteria and Bacteroidetes were observed to be significantly altered in relative abundance, but often with opposing directions of change, thus making comprehensive conclusions difficult. On the other hand, *Bifidobacteriaceae* and *Coriobacteriaceae*, both belonging to the Actinobacteria phylum, were consistently increased in relative abundance in MDD in five (Chen et al., 2018; Chung et al., 2019; Lai et al., 2019; Rong et al., 2019; Zheng et al., 2020) and four (Chen et al., 2018, 2020; Rong et al., 2019; Zheng et al., 2016) studies, respectively. Three bacterial genera, belonging to either the *Bifidobacteriaceae* or the *Coriobacteriaceae* family, were also increased in relative abundance in patients; *Eggerthella* in six studies (Chen et al., 2018, 2020; Chung et al., 2019; Lai et al., 2019; Naseribafrouei et al., 2014; Rong et al., 2019) and *Atopobium* (Chen et al., 2018; Lai et al., 2019; Rong et al., 2019) and *Bifidobacteria* (Chen et al., 2018; Chung et al., 2019; Lai et al., 2019; Rong et al., 2019) in four study populations. Four of these studies were conducted in the People's Republic of China or the Republic of China and had similar study designs. There was a decreased relative abundance of one taxa belonging to the Firmicutes phylum, *Faecalibacterium*, in seven studies (Chen et al., 2018, 2020; Jiang et al., 2015; Lai et al., 2019; Rong et al., 2019; Stevens et al., 2020; Zheng et al., 2016, 2020), but their study designs were not similar. For the remaining taxa showing differences between patients with MDD and controls, no similarities were found between studies; taxa were often observed to be increased in one study, but decreased in another, with most taxa only showing significant differences between groups in one or two studies. This suggests that these singular observations may be unique to their respective study populations, and not applicable to gut microbiota variations associated with MDD on a global scale. Opposite the other studies, Aizawa et al. (Aizawa et al., 2016) used targeted qRT‐PCR and discovered less *Bifidobacterium* counts in their patient group. Mason et al. did not find any association between specific bacteria and MDD diagnosis using qPCR, but only between the *Clostridium* cluster IV and the severity of depression (Mason et al., 2020).

Overall, the gut microbiota composition of patients and controls clustered separately in two‐thirds of the studies. Additionally, the relative abundance of *Eggerthella, Atopobium*, and *Bifidobacterium* was increased, while it was decreased for *Faecalibacterium* in a subset of studies.

## 4 DISCUSSION

Of the seventeen included studies, sixteen observed significant differences in gut microbiota composition between patients with MDD and healthy controls. An increase in relative abundance of *Eggerthella*, *Atopobium*, and *Bifidobacterium* and a decrease in relative abundance of *Faecalibacterium* was a frequent finding. Despite methodological heterogeneity, it was possible to distinguish between patients with MDD and healthy controls in almost all of the included studies based on either α‐ or β‐diversity. These results were based on highly heterogeneous study designs, with various study populations, clinical assessments, and experimental setups.

Bacterial taxa increased in relative abundance may affect signaling pathways in MDD such as bile acids signaling in the brain. Some strains of *Eggerthella* can facilitate bile acid oxidation (Harris et al., 2018), while *Bifidobacteria* can hydrolyse bile salts (Kumar et al., 2006). This was supported by the study by Chung et al., which showed perturbed bile acid metabolism in patients and a positive correlation between the relative abundance of *Eggerthella* and Beck Depression Index scores (Chung et al., 2019). Increased bile acid stimulation of the farnesoid X receptor inhibits the production of BDNF (Huang, Wang, Hu, Wang, et al., 2016). This neural growth hormone has been observed to be decreased in MDD (Dwivedi, 2009), which was also observed by Jiang et al. (Jiang et al., 2015), linking gut microbiota alterations to bile acid metabolism and symptoms in patients with MDD.

In contrast to increases in relative abundance, a loss of relative abundance of *Faecalibacterium* was reported in seven studies. The study by Jiang et al. reported that levels of *Faecalibacterium* correlated negatively with severity of depressive symptoms (Jiang et al., 2015). Lack of *Faecalibacterium* in patients with MDD may exacerbate chronic low‐grade inflammation associated with the disorder (Beurel & Lowell, 2017; Furtado & Katzman, 2015; Otte et al., 2016). This was supported by the studies by Huang et al. and Stevens et al., whom found elevated genetic pathways for LPS metabolism in patients with MDD, in addition to loss of *Faecalibacterium* (Huang et al., 2018; Stevens et al., 2020). This taxa is known to have anti‐inflammatory properties (Quevrain et al., 2016; Sokol et al., 2008). This genus produces SCFAs (Koh et al., 2016), which downregulate the production of pro‐inflammatory cytokines (Lopez‐Siles et al., 2017). The study by Kelly et al. found increased pro‐inflammatory cytokines as well as a loss of the genus *Prevotella* (Kelly et al., 2016), another known producer of SCFA (Chen et al., 2017). This indicates that loss of SCFA producers may lead to loss of regulatory interactions with the immune system. This is supported by clinical studies where supplementation with probiotics, which produce SCFAs, reduced depressive symptoms in patients with MDD (Huang et al., 2016), presumably caused by limiting low‐grade inflammation (Jakobsen et al., 2017; Park et al., 2018; Yu et al., 2017).

Overall, changes in these genera may be involved in the pathology of MDD. These changes were not observed across all studies in this review, and interpretation of the results should thus be made with caution. Despite heterogeneity concerning changes in taxa, twelve out of eighteen studies were able to distinguish patients from healthy controls based on β‐diversity. This suggests that the gut microbiota as a whole, rather than singular bacterial taxa, differentiates patients from healthy controls. Reasons for the heterogeneity in results may lie in the variations in study design and populations.

### 4.1 Standardization of study populations and applied methods may limit heterogeneity

Several factors may affect the results of different analyses in studies on gut microbiota. These include demographic variations in study populations, clinical assessment of MDD, and the experimental setup, such as bacterial nucleotide purification and 16S rRNA gene primer design.

Firstly, it is well established that dietary, geographical, and cultural impacts influence the stability, functionality, and structure of the bacterial communities (Conlon & Bird, 2014; Singh et al., 2017; Yatsunenko et al., 2012). This might explain the consistent increase in the relative abundance of the three genera *Eggerthella, Atopobium*, and *Bifidobacteria* observed in studies based in either the Peoples Republic of China (Chen et al., 2018; Lai et al., 2019; Rong et al., 2019) or the Republic of China (Chung et al., 2019). These countries have similar ethnic populations and dietary preferences, which may explain similar observations in bacterial alterations. Moreover, previous demographic studies have reported diet to influence gut microbiota composition to a greater extent than ethnic background (Khine et al., 2019). As the remaining included studies were from non‐Asian countries such as Ireland (Kelly et al., 2016), Norway (Naseribafrouei et al., 2014), the United States (Mason et al., 2020), and Denmark (Vinberg et al., 2019), regional dietary preferences may have obscured similarities in gut microbiota composition between ethnic groups. Furthermore, dietary improvements in MDD have been associated with symptom relief in a meta‐analysis of intervention studies (Firth et al., 2019), which indicates that gut microbiota alterations and MDD symptoms may be associated with dietary patterns rather than causal mechanisms.

Secondly, heterogeneity in clinical characteristics of patients may have resulted in gut microbiota composition differences between studies. The studies in this review focused on depression, but the diagnostic criteria used to determine the diagnosis differed between studies. For example, assessment of bipolar disorder was only performed in some studies (Chen et al., 2018, 2020; Chung et al., 2019; Jiang et al., 2015; Lai et al., 2019; Mason et al., 2020; Rong et al., 2019; Zheng et al., 2020). Interestingly, the study by Zheng et al. (2020) also examined patients with bipolar disorder, and found that patients with bipolar disorder and depression, respectively, were distinguishable based on gut microbial composition (Zheng et al., 2020). Likewise, comorbid inflammatory bowel disorder only led to exclusion in some studies (Chen et al., 2018; Huang et al., 2018; Jiang et al., 2015; Kelly et al., 2016; Vinberg et al., 2019; Zheng et al., 2020). Heterogeneity in patient characteristics may therefore have led to differences in gut microbiota composition between studies. The active pharmaceutical treatment can also have affected the gut microbiota composition in patients. Nonantimicrobial drugs have the potential to either influence the bacterial composition (Maier et al., 2018; Vich Vila et al., 2020), or be metabolized into bioactive compounds (Enright et al., 2016; Li et al., 2016). Additionally, different types of antidepressants may give rise to different side effects such as altered appetite with subsequent diet and weight changes (Fava, 2000; Lee et al., 2016), thereby affecting the gut microbiota. Furthermore, antidepressant drugs have documented antimicrobial properties (Vich Vila et al., 2020) specifically on gram‐positive bacteria (Macedo et al., 2017). The studies by Jiang et al. and Kelly et al. reported that use of specific selective serotonin reuptake inhibitors reduced relative abundance of *Dialister*, a gram‐positive bacterium (Jiang et al., 2015; Kelly et al., 2016). On the other hand, Chen et al. and Huang et al. only agreed on a reduction of *Ruminococcaceae* in their treatment‐naïve patients compared with the controls (Chen et al., 2018; Huang et al., 2018). In contrast, Liu et al. found elevated *Ruminoccaceae* in their cohort of patients using antidepressants, suggesting that antidepressant treatment is not solely responsible for the observed changes.

Thirdly, the DNA purification method, choice of primer pairs used for amplification, and database used for taxonomic assignment have been shown to affect experimental outcomes (Costea et al., 2017; Voigt et al., 2015). However, it was not obvious if the choice of method affected the observed variability in bacterial compositions. Two studies applied the same laboratory setup and observed an increase in relative abundance of *Eggerthella*, *Atopobium* and *Bifidobacterium* (Lai et al., 2019; Rong et al., 2019). Three additional studies also had the exact same setup for characterization of gut microbiota composition (Chen et al., 2018, 2020; Zheng et al., 2016), but they only agreed upon an increase in relative abundance of *Lachnospiraceae*. Despite their similar molecular approaches, the discrepancies suggest that the choice of patient population may affect the gut microbiota composition to a greater extent than the DNA purification method and sequencing platform.

Fourthly, the choice of hypervariable regions of the 16S rRNA gene is particularly important, as different primer pairs have been shown to induce selective bias in the detection of bacteria (Albertsen et al., 2015; Hamady & Knight, 2009; Lozupone et al., 2013). Notably, studies using the 314F/805R primer pair did not consistently observe the same differences; they did, however, agree on a reduction in relative abundance of *Faecalibacterium* (Huang et al., 2018; Stevens et al., 2020). Additionally, the 16S rRNA gene sequencing method has limitations, as the 97% clustering of OTUs generally only allows taxonomic assignment at genus level (Poretsky et al., 2014). This lack of sensitivity and specificity may account for the contrasting directions of change observed across studies as alterations at species level may obscure observations at genus level. Two studies performed shotgun metagenomics and found that species belonging to the *Bifidobacterium* genus, such as *B*. *longum* and *B*. *dentium* were increased in relative abundance in patients with MDD. This is in accordance with the other studies who observed a relative increase in *Bifidobacterium* at genus level, further strengthening the association between this genus and MDD.

## 5 CONCLUSION

Sixteen out of seventeen studies reported a difference in the gut microbiota composition between patients and controls. Several studies found either a higher relative abundance of *Eggerthella*, *Atopobium* and/or *Bifidobacterium*, or a lower relative abundance of *Faecalibacterium* or *Dialister* in patients with MDD. However, there was limited agreement between the studies, possibly due to heterogeneity in the experimental design.

## 6 SUMMATIONS

Gut microbiota was observed significantly different in most of the included studies.

*Eggerthella* was increased while *Faecalibacterium* was decreased in patients with MDD.

Variability in methodology makes generalization across studies difficult.

## 7 LIMITATIONS

This systematic review has some limitations. It was not possible to perform a meta‐analysis due to laboratory methodological heterogeneity between the studies. We did not perform a quality assessment of the studies, but relied on the studies meeting eligibility criteria. Publication bias was not investigated on the same grounds, resulting in a lack of statistical approximations.

### PEER REVIEW

The peer review history for this article is available at https://publons.com/publon/10.1002/brb3.2177.

## Supporting information

Supplementary Material

Supplementary Material

## Data Availability

There were no new data generated in this study, as all data presented were extracted from the included articles.
