# Supplementary material for: Gut microbiota variations in patients diagnosed with major depressive disorder—A systematic review
Source: Brain Behav. 2021 May 28;11(7):e02177. doi: 10.1002/brb3.2177 (PMC8323045; doi:10.1002/brb3.2177)
Supplement: Supplementary file 1 — Supplementary Material [file BRB3-11-e02177-s002.docx]

# **Supplementary Material 1**

The search was conducted on November 13^th^ using three databases; PubMed, Embase and PsychINO.

The search strategies are described below;

Database: Pubmed search strings

(((("mood disorders"[MeSH Terms] OR mood disorder[Text Word])) OR ("depressive disorder"[MeSH Terms] OR "depression"[MeSH Terms] OR depression[Text Word]))) AND ((((("microbiota"[MeSH Terms] OR microbiota[Text Word])) OR ("fecal microbiota transplantation"[MeSH Terms] OR fecal microbiota transplantation[Text Word])) OR microbiome[Text Word]) OR "brain gut axis"[All Fields]).

Database: Embase <1974 to 2020 Week 46>

Search Strategy:

--------------------------------------------------------------------------------

1 exp mood disorder/ (536354)

2 mood disorder*.mp. (59009)

3 depressi*.mp. (730993)

4 1 or 2 or 3 (806592)

5 exp microflora/ (130859)

6 fecal microbiota transplantation/ (4621)

7 brain gut axis.mp. (1200)

8 microbiome.mp. (42028)

9 microbiota.mp. (67148)

10 5 or 6 or 7 or 8 or 9 (151670)

11 4 and 10 (2479)

12 remove duplicates from 11 (2453)

13 limit 12 to dc=18000101-20201113 (2287)

Database: PsycINFO <1806 to November Week 3 2020>

Search Strategy:

--------------------------------------------------------------------------------

1 exp affective disorders/ (147813)

2 mood disorder*.mp. (23418)

3 depressi*.mp. (367524)

4 1 or 2 or 3 (381677)

5 microbiota.mp. (1074)

6 microbiome.mp. (709)

7 brain gut axis.mp. (130)

8 5 or 6 or 7 (1479)

9 4 and 8 (396)
